# Ensemble Models Predict Invasive Bee Habitat Suitability Will Expand under Future Climate Scenarios in Hawai’i

**DOI:** 10.3390/insects12050443

**Published:** 2021-05-13

**Authors:** Jesse A. Tabor, Jonathan B. Koch

**Affiliations:** 1 Department of Geography & Environmental Studies, University of Hawai’i, 200 W. Kāwili Street, Hilo, HI 96720, USA; jesseat@aggiemail.usu.edu; 2 Department of Biology, Utah State University, 5305 Old Main Hill, Logan, UT 84322, USA; 3 Tropical Conservation Biology & Environmental Science Graduate Program, University of Hawai’i, Hilo, 200 W. Kāwili Street, Hilo, HI 96720, USA; 4 Pollinating Insect—Biology, Management, and Systematics Research Unit, U.S. Department of Agriculture—Agricultural Research Service, 1410 N. 800 E., Logan, UT 84341, USA

**Keywords:** invasive, climate change, species distribution models, oceanic island, *Hylaeus*

## Abstract

**Simple Summary:**

Climate change exacerbates the threat of biological invasions by increasing climatically suitable regions for species to invade outside of their native range. Island ecosystems may be particularly sensitive to the synergistic effects of climate change and biological invasions. In Hawai’i there are 21 non-native bees that have the capacity to spread pathogens and compete for resources with native bees. We performed an ensemble of species distribution models (SDM) for eight non-native bee species (Hymenoptera: Anthophila) in Hawai’i to predict climatically suitable niches across current and future climate scenarios. We found a significant difference in habitat suitability between SDMs that were constructed with specimen records from their native and non-native (Hawai’i) range. Although SDMs predict expansion of suitable habitat into higher elevations under 2070 climate scenarios, species-rich areas are predicted to stay below 500 m elevation. Our models can inform decisions on the management of non-native bees in Hawai’i by assessing risk of invasion into new areas around the archipelago.

**Abstract:**

Climate change is predicted to increase the risk of biological invasions by increasing the availability of climatically suitable regions for invasive species. Endemic species on oceanic islands are particularly sensitive to the impact of invasive species due to increased competition for shared resources and disease spread. In our study, we used an ensemble of species distribution models (SDM) to predict habitat suitability for invasive bees under current and future climate scenarios in Hawai’i. SDMs projected on the invasive range were better predicted by georeferenced records from the invasive range in comparison to invasive SDMs predicted by records from the native range. SDMs estimated that climatically suitable regions for the eight invasive bees explored in this study will expand by ~934.8% (±3.4% SE). Hotspots for the invasive bees are predicted to expand toward higher elevation regions, although suitable habitat is expected to only progress up to 500 m in elevation in 2070. Given our results, it is unlikely that invasive bees will interact directly with endemic bees found at >500 m in elevation in the future. Management and conservation plans for endemic bees may be improved by understanding how climate change may exacerbate negative interactions between invasive and endemic bee species.

## 1. Introduction

Biological invasions are one of the most severe threats to biodiversity and natural resources. Climate change is predicted to increase the risk of biological invasions by increasing climatically suitable regions for invasive species [1]. The intersection of climate change and invasive species is considered to be one of the main factors likely to impact bee diversity, together with land use change, exposure to pesticides, and pathogen spillover [2]. Climate constraints on invasive bee species, specifically temperature and precipitation patterns, may be reduced at their range limits, potentially allowing invasive species to expand beyond their current geographic range and into novel environments [3,4,5,6]. In addition to changes in geographic distribution of species, climate change has been shown to impact plant-pollinator phenology mismatch, bee genetic composition and body size, and species interactions [7,8,9,10].

**Figure 1:**
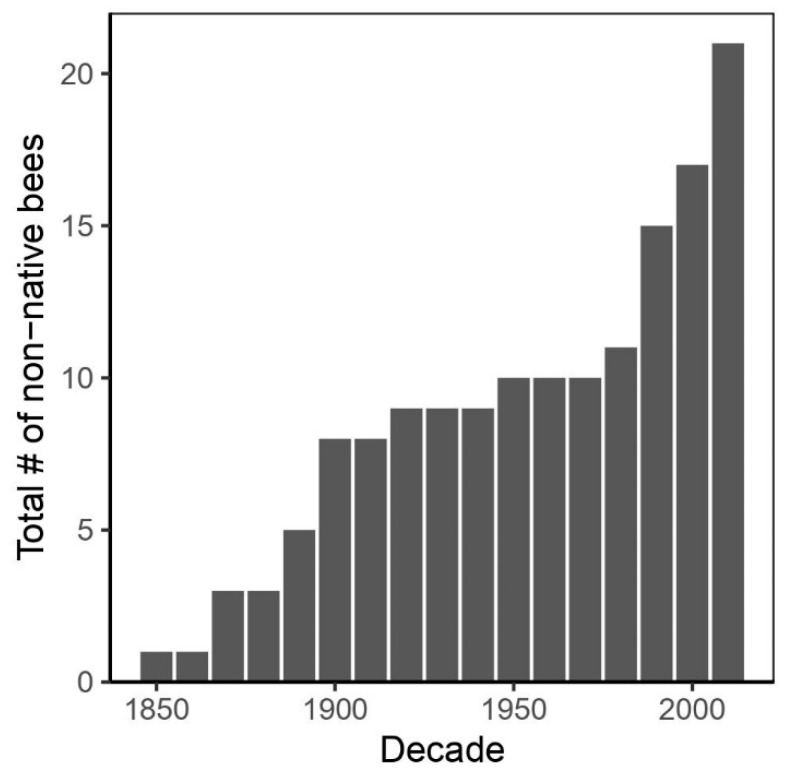
Documentation of invasive bees to Hawai’i over time. See Table 1 for the year an invasive bee species was documented in Hawai’i.

Hawai’i is one of the most remote archipelagos in the world and has been invaded by many different invasive plants and animals, including at least 21 species of introduced bees (Table 1 and Figure 1) [11,12,13,14,15]. The invasive bees present in Hawai’i have been accidentally introduced, excluding *Apis mellifera* Linnaeus, 1758, which was brought to Hawai’i in 1857 primarily to deliver pollination services to non-native *Prosopis pallida* (Fabaceae) to support the cattle industry, and subsequently honey production in 1850 [16]. In addition to *A. mellifera*, two other bee species, *Hylaeus leptocephalus* (Morawitz, 1871) and *Lithurgus scabrosus* (Smith, 1859), are of European origin and are now common and widespread [13]. Eight other species, *Ceratina arizonensis* (Cockerell, 1898), *Xylocopa sonorina* (Smith, 1874), *Lasioglossum imbrex* Gibbs, 2010, *Lasioglossum impavidum* (Sandhouse, 1924), *Lasioglossum microlepoides* (Ellis, 1914), *Lasioglossum puteulanum* (Gibbs, 2009), *Megachile gentilis* (Cresson, 1872), and *Megachile policaris* (Say, 1831), are adventive from the western United States [13,14]. One species, *Hylaeus albonitens* (Cockerell, 1905), is from Australia [13]. Finally, nine bee species, *Ceratina smaragdula* (Fabricius, 1787), *Ceratina dentipes* (Friese, 1914), *Hylaeus strenuus* (Cameron, 1897), *Megachile chlorura* (Cockerell, 1918), *Megachile diligens* (Smith, 1879), *Megachile fullawayi* (Cockerell, 1914), *Megachile lanata* (Fabricius, 1775), *Megachile timberlakei* (Cockerell, 1920), and *Megachile umbripennis* (Smith, 1853), are from Southeast Asia and the South Pacific [11,13,17,18]. There is evidence that two species, *Megachile umbripennis* and *Megachile diligens*, were introduced by Polynesians in pre-contact times [13]; however, data to support this hypothesis are lacking. In contrast, the remaining 18 invasive bee species (excluding *A. mellifera*) have been accidentally introduced following the arrival of non-kānaka maoli to the archipelago.

Non-native bee species have the potential to become invasive pests when they cause environmental, economic, or human harm [19]. Competition for floral resources and nesting sites, alteration of pollination networks, and introductions of pathogens are all possible consequences of alien bee introductions in Hawai’i [14]. The Megachilidae family has the largest invasive bee presence on the islands, with nine species. All Megachilidae species found in Hawai’i nest in a wide array of preformed cavities, but are especially prone to nest in wood or hollow plant stems [13,20]. Most *Megachile* are leaf-cutters; they line the nest cavity and construct individual cells from leaves or flower petals they have cut [13,20,21]. *M. timberlakei*, *M. umbripennis*, and *L. scabrosus* have been suggested to have negative effects on native populations by competing for nesting sites and floral resources and altering pollination networks [20,21,22]. Five species of bees in the Apidae family, including *A. mellifera*, *C. arizonensis*, *C. smaragdula*, and *C. dentipes*, have been suggested to compete for floral resources with *Hylaeus* bees in Hawai’i due to the similar proximity of habitat [12,23]. Furthermore, *X. sonorina* is a pollinator of invasive weeds [15,24,25].

Four species from the Halictidae family have been recorded in Hawai’i. In 2013, two species, *L. microlepoides* and *L. imbrex*, were frequently documented throughout the disturbed coastal and lowland areas around O‘ahu [23]. Given their dominance in disturbed habitats, both species are suggested to be significant pollinators of invasive weeds [23]. *L. impavidum* recorded in Hawai’i, has been suggested to compete for floral resources with native species because of its abundant occurrence in company with *Hylaeus* [12,13]. In California, its presumed origin, *L. impavidum* is primarily a lowland species, residing in elevations below 600 m [13]. However, its Hawai’i range has been documented at coastal localities to as high as 2700 m [13]. Finally, three invasive *Hylaeus* bees in the Colletidae family are present in Hawai’i. *H. albonitens* and *H. strenuus* are both suggested to compete for floral resources with native *Hylaeus* bees [12]. *H. leptocephalus,* which is present in Honolulu, O‘ahu, is thought to persist in low numbers only in urban sites, without spreading into native habitat [11]. As of the writing of this manuscript, no bees from the bee families Andrenidae, Stenotridae, or Melittidae have been documented in Hawai’i.

Research on how climate change could affect bees is limited for tropical island areas [1,26,27,28,29,30,31]. However, identifying the intersection between climate change and invasive species range expansion is critical for informing the management of imperiled and/or endemic biodiversity. For example, in Hawai’i there are more than 60 species of *Hylaeus* endemic to one or multiple islands [12,32]. Seven species of *Hylaeus* have been placed under the protection of the U.S. Endangered Species Act [33]. However, nearly half of the *Hylaeus* species endemic to Hawai’i are threatened due to habitat loss or alteration [12]. In fact, 10 species of *Hylaeus* may have gone extinct, as they have not been documented for a significant amount of time [12,23]. Endemic *Hylaeus* are especially vulnerable to the impacts of climate-induced biological invasions because they have fewer opportunities to adapt by altering their distribution [31,33]. The smaller land area of islands generally translates into very small populations and ranges for endemic insects such as *Hylaeus* [31,34,35,36]. Additionally, because of the limited area, *Hylaeus* endemic to Hawai’i are more vulnerable to invasive species through competition, predation, and disease spread [18,36,37]. These bees may be particularly susceptible to invasion because they rely on only a few plant species from which they gather pollen [38,39,40]. Low genetic variation, small colonizing populations, and reduced species richness may limit insular bee species in their ability to adapt [41,42,43,44]. Island species have evolved with few others and have developed survival strategies based on mutualism rather than defense mechanisms against predators and competitors [14,44]. As a result, harmful effects from climate-exacerbated biological invasions can encompass the entire habitat of endemic *Hylaeus* more readily than a continental species habitat.

The purpose of this study was to perform a comprehensive assessment of the specific regional niches of invasive bee species in Hawai’i and assess their potential expansion in the islands based on current and future climate scenarios. Little is known about potential habitat expansion of invasive bees due to climate change in Hawai’i [17]. An ability to accurately predict the impacts of climate change on species distribution is necessary to make informed decisions for biodiversity conservation. We chose to use an ensemble of species distribution models to accomplish our research aims. Our first objective in this study was to identify the bioclimatic niche for eight invasive bees in Hawai’i based on locality records from their native range and their invasive Hawai’i range. We predicted that the invaded Hawai’i niche will predict habitat suitability differently when compared to a model that projects habitat suitability based on the respective bee’s native niche. Our hypothesis was based on previous research that suggests invasive species have a high capacity to adapt to novel environments [45]. Our second objective in this study was to determine how future climate scenarios may influence the elevational distribution of invasive bees in Hawai’i. We predicted that invasive bees would expand into higher elevation habitat in future climate models. Our hypothesis was based on research that suggests species disperse to higher elevations as the temperature warms [46].

## 2. Methods

### 2.1. Species Occurrence Data

**Figure 2:**
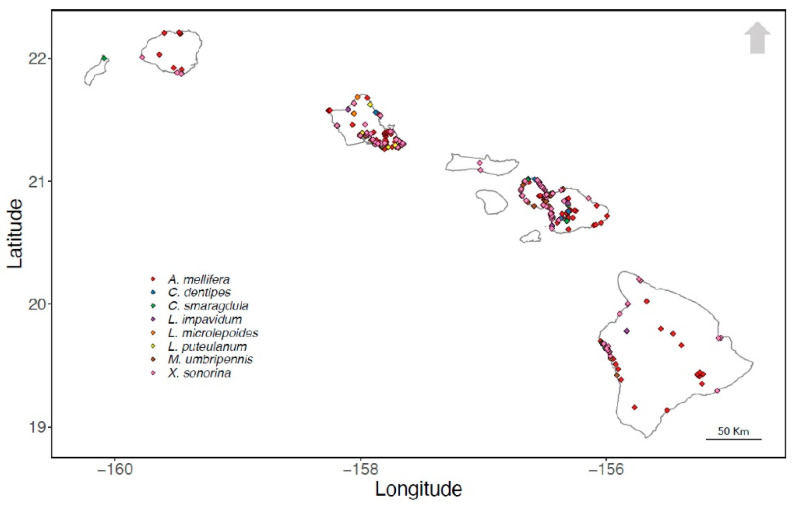
Geographic distribution of 8 invasive bees to Hawai’i.

A total of 52,511 unique locality records of invasive bees in Hawai’i were accessed from museum databases (Global Biodiversity Information Facility (GBIF; http://www.gbif.org/, Accessed: 3 June 2019) (Table S1) and Discover Life (Asher and Pickering 2011) (Table S2). The locality records, also referred to as occurrence data, were collected at different times by a diversity of collectors. All occurrence records were aggregated into 1 km^2^ cells corresponding to the resolution of environmental variables. Identical presence locations were removed and only 1 unique presence location was retained. Records were verified by published works and occurrence records that could not be validated by the literature or additional resources were excluded from the study. We selected 8 out of the total 21 species to construct species distribution models in Hawai’i: *A. mellifera*, *C. dentipes*, *C. smaragdula*, *L. impavidum*, *L. microlepoides*, *L. puteulanum*, *M. umbripennis*, and *X. sonorina* (Figure 2). Sufficiently digitized and georeferenced location information for the remaining 13 species was not publicly available for analysis via GBIF or other online resources. Data on the bees invasive to Hawai’i, including the timing of their invasion, are provided in Table 1.

### 2.2. Environmental Data

In our study, we applied 19 bioclimatic variables (derived from temperature and precipitation measures), averaged between 1970 and 2000, with a spatial resolution of 30 arcsec (~1 × 1 km), from the WorldClim 2.1 database (http://www.worldclim.org/, Accessed: 3 June 2019) [47]. The current bioclimatic variables were computed from monthly values of minimum, average, and maximum temperature and monthly precipitation. The variables used in our analysis included the following: annual mean temperature (BIO 1), mean diurnal range (mean of monthly (max temp–min temp)) (BIO 2), isothermality (BIO2/BIO7) (×100) (BIO 3), temperature seasonality (standard deviation ×100) (BIO 4), max temperature of warmest month (BIO 5), min temperature of coldest month (BIO 6), temperature annual range (BIO 5–BIO 6) (BIO 7), mean temperature of wettest quarter (BIO 8), mean temperature of driest quarter (BIO 9), mean temperature of warmest quarter (BIO 10), mean temperature of coldest quarter (BIO 11), annual precipitation (BIO 12), precipitation of wettest month (BIO 13), precipitation of driest month (BIO 14), precipitation seasonality (coefficient of variation) (BIO 15), precipitation of wettest quarter (BIO 16), precipitation of driest quarter (BIO 17), precipitation of warmest quarter (BIO 18), and precipitation of coldest quarter (BIO 19). To reduce multicollinearity among the environmental variables, a principal component analysis was conducted to highlight the relationship between the target species occurrences and the specific environmental combinations within the archipelago. Variables were chosen based on orthogonal direction and overall environmental variation following strategies implemented in the BIOMOD2 package [48].

Distributions of the 8 invasive bee species were also modeled for future climatic conditions. We used projected bioclimatic variables for the period 2070 from representative greenhouse gas concentration pathway (RCP) scenario, RCP 8.5. RCP 8.5, derived from the Coupled Model Intercomparison Project Phase 5 (CMIP5), represents a radiative forcing of +8.5 W/m for the period 2000–2100, predicted to raise the average temperature 4.3 °C by 2100 [49]. Many research groups around the world have produced different global climate models (GCMs), which have been submitted to the CMIP5. GCMs can be used to forecast climate change because they capture the processes that respond to climate forcing. The bioclimatic variables used for modeling current distribution were used to predict future distribution of the invasive bees. We downloaded future bioclimate data of 12 GCMs: BCC-CSM1-1, CCSM4, GFDL-ESM2G, GISS-E2-R, HadGEM2-AO, HadGEM2-ES, IPSL-CM5A-LR, MIROC-ESM-CHEM, MIROCESM, MIROC5, MRI-CGCM3, and NorESM1-M from the WorldClim 1.2 database. We created an ensemble of the 12 GCMs by taking average values and used the ensemble values as predictors. Our multi-model ensemble average accounts for inherent variability among the different future climate GCMs.

### 2.3. Species Distribution Modeling

Species distribution models (SDM) have been used to compare species’ regional ecological niches and forecast the range shifts of species under future climate change scenarios [1,50,51,52,53]. Species distribution modeling is an approach that predicts the distribution of a species across geographic space and time using the correlation between the geographic occurrence or abundance of a species and corresponding environmental conditions to predict the most suitable habitat [52,54]. Various methods have been used in SDMs, including regression, machine learning, classification, and maximum entropy [48,55]. Discrepancies between different techniques can be large and performance can vary significantly across different algorithms. Considering the variability between algorithms, we chose to use an ensemble modeling approach. Ensemble modeling of species distributions involves simulations across more than 1 set of initial conditions, model classes, model parameters, and boundary conditions by combining individual SDMs built through different modeling algorithms [55]. By using a wide range of approaches to test the models, ensemble modeling accounts for inter-model variability and uncertainties in predictions [56]. However, prediction uncertainty may also be dependent on GCM and RCP variation [55].

The analysis was conducted in R environment v 3.6.1 (R Core Team, 2019) using the BIOMOD2 package (Grenoble, France) [48]. The algorithms used to produce an ensemble model were as follows: 3 regression methods (GAM: general additive model; GLM: general linear model; and MARS: multivariate adaptive regression splines), 3 machine learning methods (ANN: artificial neural network; GBM: generalized boosting model; and RF: random forest), 2 classification methods (CTA: classification tree analysis; FDA: flexible discriminant analysis), and 1 maximum entropy approach (MAXENT) [57]. To identify the differences between bioclimatic niche of native range and Hawai’i range, 2 independent approaches were used: 1 approach using native range occurrences to predict favorable areas in Hawai’i and 1 approach using Hawai’i range occurrences to predict favorable areas in the Hawai’i. As these models required background data and the actual absence data were unavailable, we used 10,000 pseudo-absences randomly generated in the native range environmental space and 7000 pseudo-absences randomly generated in the Hawai’i environmental space. The models were calibrated by using 80% of the occurrence points (presence and pseudo-absence) as training data and evaluated by using the remaining 20% as testing data [46]. We repeated the process of pseudo-absence generation 3 times and repeated evaluation runs 4 times per species, resulting in a total of 108 models per species (9 models, 4 evaluation runs, and 3 pseudo-absence selection procedures) under each climate scenario (i.e., current climate (1970–2000) and RCP 8.5 (2070)).

The area under the curve (AUC) of receiver operating characteristics and true skills statistics (TSS) were used to measure model validation and predictive performance. The AUC value represents the predictive power of a model [58]. According to the AUC value, the model was graded as “poor” (if AUC = 0.6–0.7), “fair” (AUC = 0.7–0.8), “good” (AUC = 0.8–0.9), or “excellent” (AUC = 0.9–1.0) [58]. TSS measure ranges from −1 to +1 where +1 indicates a perfect agreement, and a TSS value below 0.4 indicates poor model discrimination [58]. From the 108 models per species, we built ensemble models using a weighted-mean approach in which weights are awarded for each model proportionally to their evaluation metrics scores. Only the models with greater than fair predictive accuracy (TSS > 0.5) to greater than good predictive accuracy (TSS > 0.8) were used to build an ensemble from the projection outputs [4,48].

### 2.4. Bioclimatic Niche Analysis

Binary maps (suitable and unsuitable) were produced using the optimal threshold that maximizes the TSS score as a cut-off value using the *Biomod_RangeSize* function, which then converted the projected occurrence probabilities during the cross-validation procedure. These binary maps were used to measure the loss, stability, and gain of predicted suitable areas following the predicted climate scenario demonstrated with an RCP 8.5 in 2070. From these binary maps, we measured the range size of the studied invasive species as represented by the number of climatically suitable pixels across Hawai’i for the designated time period (Table 2).

We calculated niche similarity between the native and the invasive Hawai’i range using the bioclimatic variables selected from the principal component analysis [48]. Native models were calibrated using the native range and projected onto the invasive Hawai’i environment. Alternatively, invasive Hawai’i models were calibrated using the occurrence records from the invasive Hawai’i range and projected onto the Hawai’i environment. We extracted the model values and tested for differences in habitat suitability between the native and invasive Hawai’i range with a Wilcoxon test across all 8 species.

### 2.5. Species Richness Analysis

To determine how projected climate change will impact invasive species richness, we conducted a species richness analysis to identify the regions potentially suitable for the maximum number of invasive bees under current and future climate. We combined binary maps of climatically suitable niches for all 8 species to generate species richness (cells with a higher value indicating high species diversity) and extent maps (cells occupied by at least a single species). We calculated changes in areas of both richness and extent of potentially suitable regions under current and future climate. Using a digital elevation model accessed from the University of Hawai’i at Manoa School of Ocean and Earth Science Technology (http://www.soest.hawaii.edu/coasts/data/hawaii/dem.html, Accessed: 3 June 2019) combined with the species richness maps, we determined how future climate scenarios may influence the elevational distribution of invasive bees in Hawai’i. Finally, we tested for the effect of species richness (i.e., 1–8 species) and climate timeframe (i.e., contemporary vs. future) on the elevation distribution of species richness with a non-parametric analogue to the two-way ANOVA, the Scheirer-Ray-Hare test.

## 3. Results

### 3.1. Species Occurrence Data

As publicly available and georeferenced data was lacking for the majority of the invasive bees to Hawai’i, we were able to pursue our research objectives with 8 of 21 invasive bee species: *A. mellifera*, *C. dentipes*, *C. smaragdula*, *L. impavidum*, *L. microlepoides, L. puteulanum*, *M. umbripennis*, and *X. sonorina*. These species represent bees that have georeferenced data in both their native and invasive Hawai’i geographies. We summarize the data in Table 1 and provide the citation for the data in Tables S1 and S2.

### 3.2. Species Distribution Modeling

**Figure 3:**
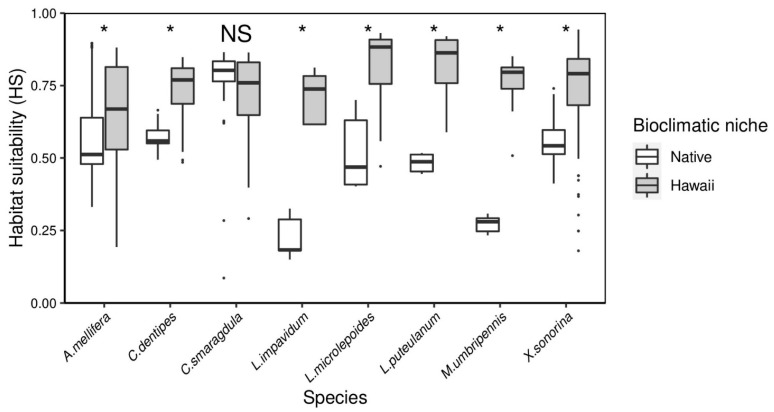
Estimates of habitat suitability (HS) using native and invasive to Hawai’i georeferenced records in species distribution models. Species with asterisk represents a significant difference in HS. Species with NS represents no significant difference in HS.

Based on AUC and TSS metrics, the invasive Hawai’i niche, calibrated from the Hawai’i range occurrences, predicted higher habitat suitability than records sampled from their respective native geographies (Figure 3). Due to the native occurrence model poorly predicting actual species occurrences in Hawai’i, we decided to use the invasive Hawai’i SDMs to project habitat suitability in the future. The model performance was evaluated by the scores of two (AUC and TSS) performance metrics (Table 3). The average AUC values of the eight studied species ranged from 0.937 (*A. mellifera*) to 0.998 (*L. puteulanum* and *M. umbripennis*), indicating that the models have excellent predictive accuracy. Likewise, the average TSS value ranged from 0.731 (*A. mellifera*) to 0.991 (*L. puteulanum*), indicating good predictive accuracy. Moreover, we only used the models with highest predictive accuracy to build an ensemble from the projection outputs (Table 4).

### 3.3. Bioclimatic Niche Analysis

Following our PCA approach to selecting variables to construct SDMs, we identified a combination of four from a pool of seven bioclimatic variables to construct the final SDMs for each of the eight species: BIO 1, BIO 2, BIO 7, BIO 9, BIO 12, BIO 15, and BIO 19. Based on pairwise analyses of SDMs, our results indicated little overlap between the native niche and the invasive Hawai’i niche across seven species (*A. mellifera*: *W* = 8452.5, *p* = 2.65 × 10^−5^, *C. dentipes*: *W* = 166, *p* = 7.80 × 10^−9^, *L. impavidum*: *W* = 0, *p* = 0.00793, *L. microlepoides*: *W* = 28, *p* = 5.34 × 10^−7^, *L. puteulanum*: *W* = 0, *p* = 3.18 × 10^−7^, *M. umbripennis*: *W* = 0, *p* = 1.29 × 10^−8^, *X. sonorina*: *W* = 849, *p* = 0.0193) (Figure 3). Our analyses found that the only niche overlap between the native and Hawai’i ranges occurred across the *C. smaragdula* distribution (*W* = 974.5, *p* = 0.09402).

### 3.4. Species Richness Analysis

**Figure 4:**
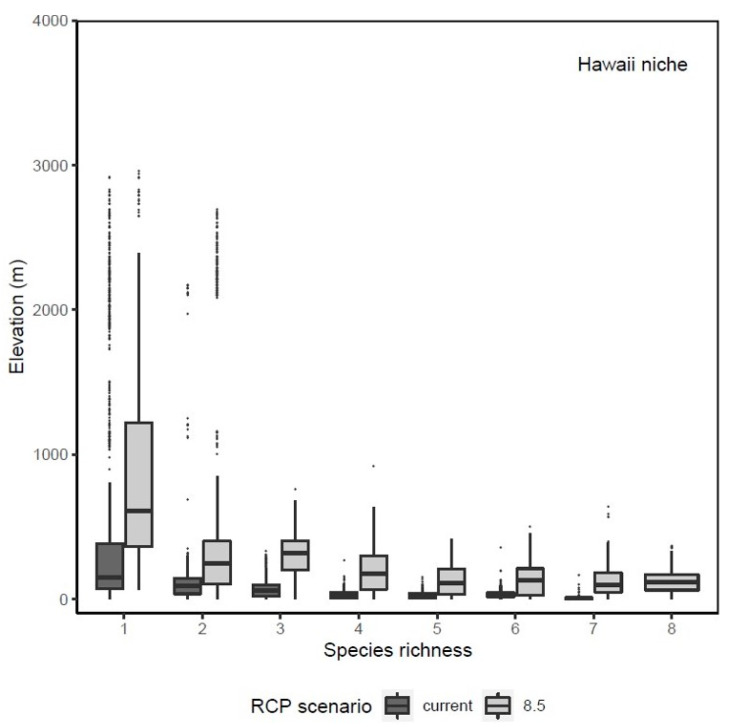
Elevation distribution of species richness across current and future (i.e., 8.5) climate scenarios for invasive to Hawai’i models. Black line in each boxplot represents the median elevation distribution.

**Figure 5:**
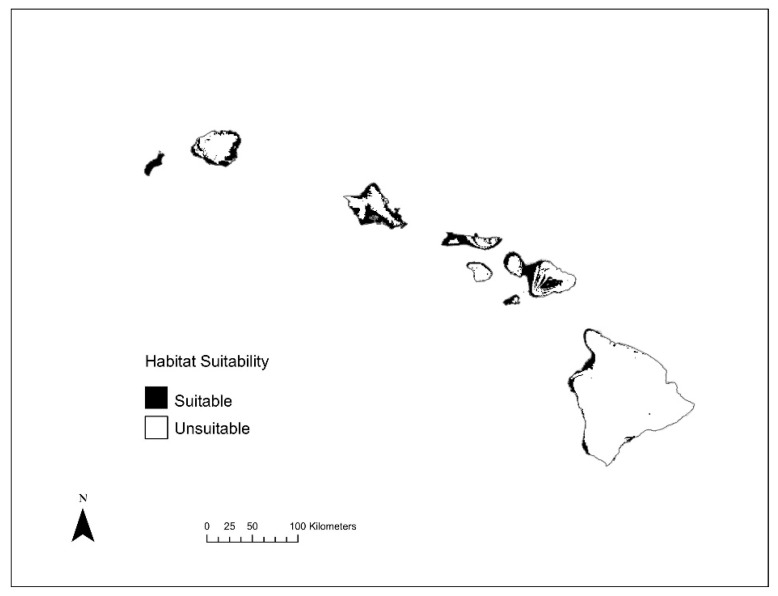

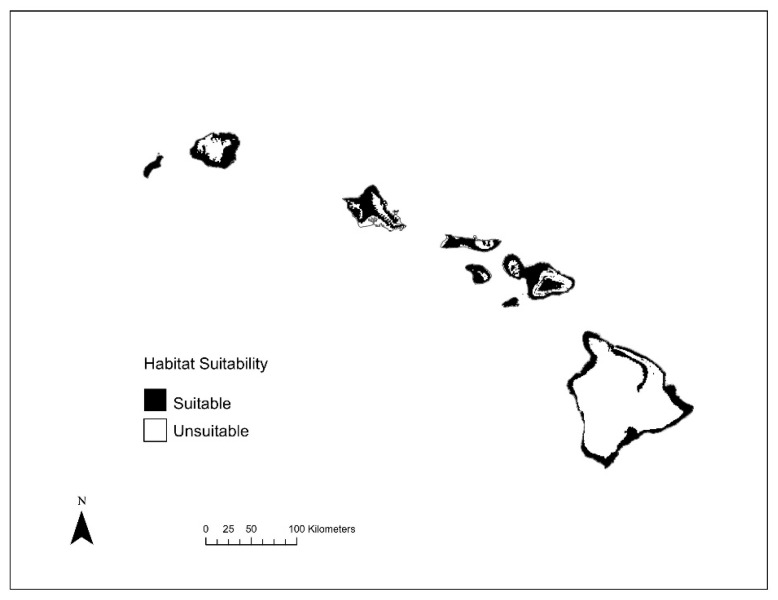
Habitat extent for all 8 invasive bee species across current (**top**) and future (**bottom**) climate scenarios for invasive to Hawai’i models.

**Figure 6:**
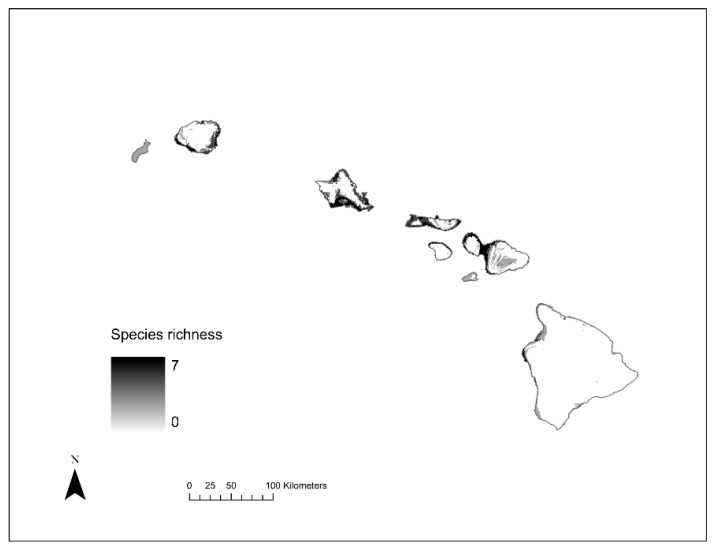

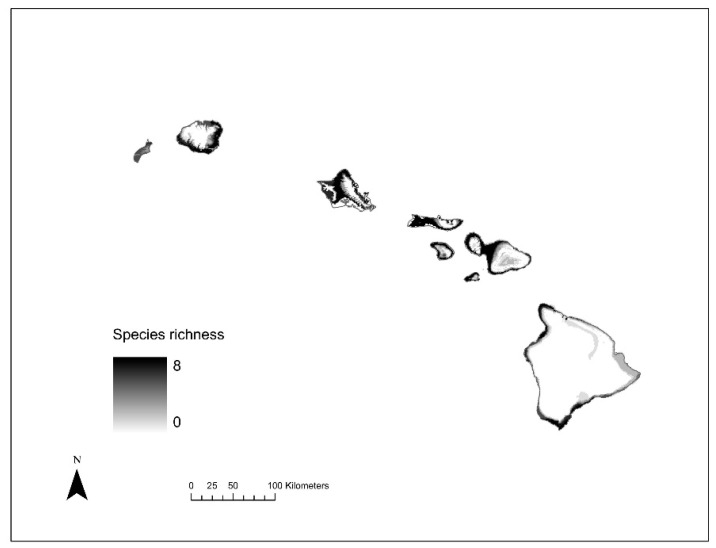
Species richness (i.e., 1–8) across current (**top**) and future (**bottom**) climate scenarios for invasive to Hawai’i models.

Based on the invasive Hawai’i SDMs, areas of potentially suitable niches for the studied species mostly occupy low elevation areas (Figure 4). Out of the eight species, five had potentially suitable areas that covered less than 5% of land area under current climate in Hawai’i (Table 2). Specifically, *C*. *dentipes*, *L*. *imbrex*, *L*. *microlepoides*, *L*. *puteulanum*, and *M*. *umbripennis* are predicted to have a restricted distribution under current climate conditions, whereas *A*. *mellifera*, *C*. *smaragdula*, and *X*. *sonorina* have a wider distribution under the current climatic conditions (Table 2 and Figure 5). However, under the current climate conditions, *X*. *sonorina* is predicted to have the largest suitable habitat, covering 15% of the land area in Hawai’i. The predicted suitable bioclimatic niche for *L*. *puteulanum* under current climate conditions covered only 1% of the land area in Hawai’i. Furthermore, under current climate conditions, the SDMs predicted most of the suitable habitat under 500 m with most areas occupied by a single species (Figure 4 and Figure 6).

Our analysis identified that “hotspot areas” of high invasive species richness are distributed primarily in low elevation sites (<500 m) under current and future climate conditions. Specifically, Scheirer-Ray-Hare tests found a significant difference in elevational distribution of species richness across current and future climate scenarios (Climate (current vs. 2070): *H* = 803.8, *df* = 1, *p* = 0; Species Richness (SR): *H* = 3828.2, *df* = 7, *p* = 0; Climate: SR: *H* = 57.5, *df* = 6, *p* = 1.45 × 10^−10^) (Figure 4), with highest median species richness of invasive species to be present in habitats at low elevation sites (<500 m). While our analyses demonstrated both gains and losses of suitable habitat when comparing contemporary and future climate models (Table 2), we predict that there will be a major geographic expansion of habitat suitability for invasive bees in Hawai’i under future climate scenarios (Figure 5 and Figure 6). Our SDMs predict that climatically suitable regions would increase on average by ~934.8% (±3.4% SE) for all species except *L. impavidum* (Table 2). For example, a maximum increase of 2425.7% habitat suitability for *L*. *microlepoides* is estimated. This habitat suitability increase is predicted to cover 25.9% of the terrestrial habitat across the archipelago (Table 2). However, the species estimated to experience the greatest range gain, in terms of geographic coverage, is *A. mellifera*. This economically significant species to Hawai’i is estimated to experience high habitat suitability across 40.3% of the terrestrial habitat across the archipelago (Table 2).

## 4. Discussion

The models developed in our study predict that the changing climate will create additional climatically suitable areas for invasive bees in Hawai’i over the next 50 years. This research provides baseline information to aid in effective management of invasive bees by showing the areas which have suitable niches for the invasive bees. Our study of eight of the 21 invasive bee species in Hawai’i found that SDMs based on georeferenced records from their invasive range were better at predicting habitat suitability in the invasive range than records found in their native range. Furthermore, while the eight invasive bees are predicted to expand in their geographic range over the next 50 years, the expansion will likely be limited to low elevation habitats <500 m, with little evidence for an expansion up to high elevation habitats (>500 m). Of all the species studied, *L*. *microlepoides* is predicted to encounter the greatest habitat suitability expansion, again primarily at low elevation habitats across the archipelago, whereas *A. mellifera* will likely be present in ~40% of the terrestrial land area of the major Hawaiian islands.

The results and approach of our study may be helpful for the prevention and early detection of invasive insects, namely bees, in determining suitable niches outside of their native range. The development of SDMs is a useful approach in determining how bees and other insects will expand outside of their native niche [1,45,59]. However, it is also clear that there are limitations to SDMs in predicting the invasive spread of bees, as evidenced by data collected during ground surveys of invasive *A. manicatum* in northeastern North America [60]. Specifically, Graham et al. [60] found no evidence for *A. manicatum* in the vast majority of areas predicted by Strange et al. [45] SDMs of *A. manicatum*—despite standardized surveys of their study area in northeast North America. However, the absence of an invasive species in a suitable area outside their native range does not necessarily suggest poor model performance, but may be an artifact of the species not yet dispersing into the new habitats, especially if they are recent invaders [61,62]. Furthermore, other limiting factors such as nesting biology, diet breadth, and phenology may also impact detection and colonization rates of invasive species, especially on oceanic islands.

The SDMs generated in this study predict that additional suitable areas for invasive bees are expected to emerge in the higher elevation zones of Hawai’i. However, our models predict that the expansion of invasive bees across elevation in Hawai’i is limited. The creation of climatically suitable regions for invasive bees in the high-elevation regions, which are already vulnerable to climate change, may have negative consequences for endemic biodiversity in the future. Therefore, biological invasions will add pressure and increase risks to the most vulnerable ecosystems in Hawai’i. Along with climate change, anthropogenic development is considered a major driver that promotes biological invasions in island ecosystems. For example, roads play an important role in the spread of alien species by facilitating dispersal corridors into the adjacent ecosystem [63]. This may promote the dispersal of invasive bees from the coast when climate change opens up suitable regions by reducing climatic barriers for them to invade high elevation regions [3]. Therefore, monitoring and management of invasive bees in Hawai’i should account for the vulnerability posed by climate change combined with anthropogenic activities. However, the suitable regions identified may not be occupied by invasive bees due to natural dispersal barriers created by lava flows, which are predominant physical features in Hawai’i.

Geographic shifts in species range involve multiple ecological processes such as dispersal, physiology, species interactions, population interactions, and evolution operating at multiple scales [64]. SDMs do not explicitly consider these inherent biological properties, which interact with ecological processes and ultimately cascade to species persistence [52]. Furthermore, there are other potential issues such as environmental variables used in the analysis, modeling algorithm, GCMs, collinearity, model complexity, model evaluation method, and threshold values to produce binary maps that can influence model outcomes. In addition, future land use (e.g., road building) change scenarios can also alter future species distributions [1,17]. Improvements of models based on natural history and ecological information and increased availability of specimen data are crucial issues for enhancing the predictive accuracy of the models [60]. 

Climate change has the potential to create more suitable regions for adventive bee species in Hawai’i. Climate change alters the distribution, composition, and phenology of native species while facilitating the dispersal of invasive species by removing current climatic barriers [3,7]. In our study, it is clear that habitat suitability for invasive bees will expand considerably in comparison to their current bioclimatic niche. However, this expansion of species richness is predicted to occur primarily below 500 m. Our results suggest that in Hawai’i, cooler temperatures associated with higher elevation sites may limit the invasion of invasive bees [4]. However, climate may not be the only limiting factor for invasive bees to spread to high elevation habitats. Thus, a better understanding of species traits, dispersal pathways, and the mechanism of the natural filters that prevent colonization of invasive species are necessary. The results of our research show a diverse set of hypothetical responses by invasive bees to climate change; therefore, species-specific prioritization exercises may be helpful to better manage and monitor specific invasive bee species.

Given the endangered status of endemic *Hylaeus* bees in Hawai’i, it is important to monitor and predict suitable habitats of invasive bees. By highlighting the similarities and differences between the native and invasive Hawai’i bioclimatic niche, results can inform stakeholders on the invasive potential of invasive bees [17,45]. Furthermore, the SDMs in our study provide evidence of current and future risks associated with invasive bee species in Hawai’i. For example, endemic *Hylaeus* bees, including species protected under the U.S. Endangered Species Act, that are distributed at low elevation habitats will likely continue to interact with invasive bees over the next 50 years. However, endemic *Hylaeus* bees at high elevation habitats will likely experience limited interactions with invasive bees. Given these predicted ecological differences, endemic *Hylaeus* bees will experience different disease and resource pressures.

While the current study used an ensemble model approach to estimate future climate models and species distribution models, the accuracy of models nevertheless relies on the accuracy of the data being used. The accuracy of GCMs is essential to predictability in SDMs, and the lack of bioclimatic agreement between different databases is an important factor [65]. Furthermore, including other bioclimatic variables in the initial model building process may produce more accurate models [66]. For example, a study that combined ENVIREM variables with Bioclim variables improved model performance in 13 out of 20 species [66]. For the current study of non-native bees in Hawai’i, model performance may be improved in future modeling exercises by including topographical variables such as slope, aspect, heat load index, and terrain ruggedness.

Many other approaches to SDMs exist that may improve or alter predictability. For example, a single source modeling approach often produces biased spatial predictions [65]. However, one study found that fine-tuned individual models may sometimes perform better than ensemble models [67]. Furthermore, Zhu et al. 2021 demonstrated that using a weighted mean approach to produce ensemble models may produce models overly influenced by the extreme values of individual predictions [67]. Sample size is also a large factor of influence on the accuracy of SDMs [68,69]. Algorithms can be fine-tuned to produce models with the highest predictability [70]. However, using an ensemble modeling approach to SDMs may overlook these fine-tuning capabilities. Many different scoring metrics exist for determining accuracy and predictability of SDMs such as the Brier score, Boyce index, or the Jaccard and Sorensen indices [4,71,72,73,74]. In this study, we chose to use AUC and TSS. While this method is widely used in ecology, some research suggests that TSS can be a misleading measure of model performance because of its dependence on prevalence [73].

In our study, we presented a geographic and bioclimatic assessment of eight invasive bees in Hawai’i. However, more research and surveys are needed to document the distribution and spread of the additional 13 bee species not included in our study. Our research has important implications for the management and monitoring of biological invasions of bees in Hawai’i. Our analysis also highlights the value of using SDMs to estimate species richness under future climate scenarios. As the climate changes, new habitats will emerge that may be suitable for adventive bee species. Climate change facilitates dispersal, introduction, and naturalization of adventive species as well as reduces the resilience of local ecosystems [75]. Thus, identifying emerging pests that may pose a threat to ecosystems in Hawai’i and the agricultural economy through SDMs is a useful tool in management and conservation.

## Figures and Tables

**Table 1:** Occurrence data download summary for 21 bees (Hymenoptera: Anthophila) that are invasive to Hawai’i from the Global Biodiversity Information Facility (GBIF) webpage (http://gbif.org, Accessed: 3 June 2019).

Family	Genus	Species	No. of Records on GBIF	No. GeoRef Records on GBIF	Final No. GeoRef Records	Year Documented in Hawai’i	General Native Range
Apidae	*Apis*	*mellifera*	150,293	134,926	50,640	1857	Europe (Snelling 2003)
Apidae	*Ceratina*	*arizonensis*	836	828	101	1950	Southwestern United States (Daly 1973)
Apidae	*Ceratina*	*smaragdula*	409	318	107	1998	Southeast Asia (Hirashima, 1969, Snelling 2003)
Apidae	*Ceratina*	*dentipes*	183	145	70	1996	Southeast Asia (Snelling 2003)
Apidae	*Xylocopa*	*sonorina*	155	138	101	1874	North America (Snelling 2003)
Colletidae	*Hylaeus*	*leptocephalus*	589	490	247	1958	Europe (Snelling 2003)
Colletidae	*Hylaeus*	*albonitens*	924	172	59	1995	Australia (Magnacca & King 2013)
Colletidae	*Hylaeus*	*strenuus*	1	1	2	2007	India (Magnacca 2011)
Halictidae	*Lasioglossum*	*imbrex*	0	0	49	2005	Western North America (Gibbs 2010)
Halictidae	*Lasioglossum*	*impavidum*	337	326	28	1994	Coastal California (Snelling 2003)
Halictidae	*Lasioglossum*	*microlepoides*	13,258	13,242	180	2010	Western North America and Northern Mexico (Magnacca & King 2013)
Halictidae	*Lasioglossum*	*puteulanum*	1752	1749	188	2012	Eastern North America (Gibbs, 2011); USGS (https://www.usgs.gov/media/images/lasioglossum-nr-puteulanum-male-side, Accessed: 3 June 2019)
Megachilidae	*Lithurgus*	*scabrosus*	36	18	14	1907	Europe (Snelling 2003)
Megachilidae	*Megachile*	*chlorura*	3	1	2	1988	Philippines (Snelling, 2003); Southeast Asia (Rasmussen 2012)
Megachilidae	*Megachile*	*diligens*	5	0	15	1879	South Pacific according to Snelling, 2003), Southeast Asia according to Rasmussen 2012
Megachilidae	*Megachile*	*fullawayi*	21	17	12	1921	Guam according to Cockerell, but likely brought from Asia. (Snelling 2003)
Megachilidae	*Megachile*	*gentilis*	731	668	230	1899	Northwestern USA, species know from southern BC (Snelling 2003); as M. palmarum Perkins
Megachilidae	*Megachile*	*lanata*	345	302	86	2012	Southeast Asia (Gonsalez et al. 2019), India (Magnacca et al. 2013)
Megachilidae	*Megachile*	*policaris*	1114	891	382	2018	Georgia and Florida, west to California and Mexico
Megachilidae	*Megachile*	*timberlakei*	10	8	8	1904	First documented in Hawai’i, probably South Pacific region. (Snelling 2003)
Megachilidae	*Megachile*	*umbripennis*	177	148	39	1898	Northern India and China (Timberlake, 1921)

**Table 2:** Predicted distribution of habitat suitability across the 8 major islands of Hawai’i under contemporary and future (2070, RCP 8.5) global climate models. Estimates presented in this table approximate 1 pixel to 1 km^2^ (30 arc sec).

Species	Current Range (km^2^)	Range Loss (km^2^) (2070, RCP 8.5)	Range Gain (km^2^) (2070, RCP 8.5)	% Loss (2070, RCP 8.5)	% Gain (km^2^) (2070, RCP 8.5)
*A. mellifera*	2562 (12%) *	168	5538 (40.3%)	6.6%	216.2%
*C. dentipes*	889 (4.4%)	21	3987 (24.3%)	2.4%	448.5%
*C. smaragdula*	748 (3.7%)	0	3600 (21.7%)	0	481.3%
*L. impavidum*	1217 (6.0%)	171	202 (7.0%)	14.1%	16.6%
*L. microlepoides*	364 (1.8%)	0	4827 (25.9%)	0	2425.7%
*L. puteulanum*	213 (1.0%)	0	4202 (22.0%)	0	1972.8%
*M. umbripennis*	377 (1.8%)	0	3308 (18.3%)	0	1769.0%
*X. sonorina*	2958 (14.7%)	13	4390 (36.6%)	0.4%	148.4%

* Percentages in parenthesis estimate percent of suitable area for 8 invasive bees relative to total estimated area of Hawai’i in the contemporary time (1970–2000) and projected into 2070 (20,061 pixels total at 30 arc sec). Percentages without parenthesis estimate percent relative to current range size.

**Table 3:** True skills statistic (TSS) and area under the curve (AUC) test statistics for 8 SDMs predicted the distribution of 8 invasive bees to Hawai’i.

Species	Invasive Model TSS	Invasive Model AUC	Native Model TSS	Native Model AUC
*A. mellifera*	0.731	0.937	0.921	0.995
*C. dentipes*	0.958	0.993	0.815	0.956
*C. smaragdula*	0.917	0.987	0.759	0.939
*L. impavidum*	0.94	0.975	0.807	0.936
*L. microlepoides*	0.983	0.997	0.602	0.869
*L. puteulanum*	0.991	0.998	0.796	0.954
*M. umbripennis*	0.983	0.998	0.907	0.976
*X. sonorina*	0.753	0.943	0.63	0.89

**Table 4:** True skills statistic (TSS) thresholds to produce ensemble models for 8 invasive bees to Hawai’i in their native range and invasive Hawai’i range.

Species	Native Model	Invasive Model
*A. mellifera*	0.8	0.5
*C. dentipes*	0.5	0.8
*C. smaragdula*	0.8	0.8
*L. impavidum*	0.5	0.5
*L. microlepoides*	0.5	0.8
*L. puteulanum*	0.5	0.8
*M. umbripennis*	0.5	0.5
*X. sonorina*	0.5	0.8

## Data Availability

Data downloaded from GBIF are described in Table S1. Scripts and data used for all analyses are available at https://github.com/jesseat12/hawaii_bee (Accessed: 8 April 2021).

## Supplementary Materials

The following are available online at https://www.mdpi.com/article/10.3390/insects12050443/s1, Table S1: Bee specimen records evaluated from the Global Biodiversity Information Facility (http://gbif.org, Accessed: 3 June 2019) and their respective DOIs across species-specific data downloads. Table S2, Bee specimen records evaluated from the American Museum of Natural History to determine their capacity for informing SDMs across their (a) native and (b) non-native (invasive) Hawai’i distribution. Not all records could be used as they did not provide publicly available georeferenced data on the website.
