# Supplementary material for: Ensemble Models Predict Invasive Bee Habitat Suitability Will Expand under Future Climate Scenarios in Hawai’i
Source: Insects. 2021 May 13;12(5):443. doi: 10.3390/insects12050443 (PMC8152285; doi:10.3390/insects12050443)
Supplement: Supplementary file 1 [file insects-12-00443-s001.zip › insects-1196676-supplementary.pdf]

Supplementary Table S1. Bee specimen records evaluated from the Global Biodiversity Information Facility (http://gbif.org) and their respective DOIs across species-specific data downloads.

Species	DOI
Apis mellifera	https://doi.org/10.15468/dl.tqfxlt
Ceratina arizonensis	https://doi.org/10.15468/dl.yk7ttv
Ceratina dentipes	https://doi.org/10.15468/dl.pcida6
Ceratina smaragdula	https://doi.org/10.15468/dl.plwhz0
Hylaeus albonitens	https://doi.org/10.15468/dl.3l2lg5
Hylaeus leptocephalus	https://doi.org/10.15468/dl.j94onx
Hylaeus strenuus	https://doi.org/10.15468/dl.nwqqf1
Lasioglossum impavidum	https://doi.org/10.15468/dl.dbnlm5
Lasioglossum microlepoides	https://doi.org/10.15468/dl.d2nnu8
Lasioglossum puteulanum	https://doi.org/10.15468/dl.lcirmt
Lithurgus scabrosus	https://doi.org/10.15468/dl.6mzy2w
Megachile chlorura	https://doi.org/10.15468/dl.62huck
Megachile diligens	https://doi.org/10.15468/dl.x3u6uh
Megachile fullawayi	https://doi.org/10.15468/dl.8geeyr
Megachile gentilis	https://doi.org/10.15468/dl.kr6itq
Megachile lantana	https://doi.org/10.15468/dl.knz6hf
Megachile policaris	https://doi.org/10.15468/dl.ebxepw
Megachile timberlakei	https://doi.org/10.15468/dl.ed7jha
Megachile umbripennis	https://doi.org/10.15468/dl.v3ld4z
Xylocopa sonorina	https://doi.org/10.15468/dl.nnr2q5

Supplementary Table S2. Bee specimen records evaluated from the American Museum of Natural History to determine their capacity for informing SDMs across their (a) native and (b) non-native Hawaiʻi distribution. Not all records could be used as they did not provide publicly available georeferenced data on the website.

(a) Native records.

ID	Species	Institution
1	*Ceratina dentipes*	American Museum of Natural History, Bee specimen record database. https://www.discoverlife.org/mp/20l?id=AMNH_BEES108761
2		American Museum of Natural History, Bee specimen record database. https://www.discoverlife.org/mp/20l?id=AMNH_BEES108731
3		American Museum of Natural History, Bee specimen record database. https://www.discoverlife.org/mp/20l?id=AMNH_BEES108727
4		American Museum of Natural History, Bee specimen record database. https://www.discoverlife.org/mp/20l?id=AMNH_BEES108738
5		American Museum of Natural History, Bee specimen record database. https://www.discoverlife.org/mp/20l?id=AMNH_BEES108737

6 American Museum of Natural History, Bee specimen record database. https://www.discoverlife.org/mp/20l?id=AMNH_BEES108792

7 American Museum of Natural History, Bee specimen record database. https://www.discoverlife.org/mp/20l?id=AMNH_BEES108794

8 American Museum of Natural History, Bee specimen record database. https://www.discoverlife.org/mp/20l?id=AMNH_BEES108795

9 American Museum of Natural History, Bee specimen record database. https://www.discoverlife.org/mp/20l?id=AMNH_BEES108791

10 American Museum of Natural History, Bee specimen record database. https://www.discoverlife.org/mp/20l?id=AMNH_BEES108730

11 American Museum of Natural History, Bee specimen record database. https://www.discoverlife.org/mp/20l?id=AMNH_BEES108729

12 American Museum of Natural History, Bee specimen record database. https://www.discoverlife.org/mp/20l?id=AMNH_BEES108790

13 American Museum of Natural History, Bee specimen record database. https://www.discoverlife.org/mp/20l?id=AMNH_BEES108787

14 American Museum of Natural History, Bee specimen record database. https://www.discoverlife.org/mp/20l?id=AMNH_BEES108789

15 American Museum of Natural History, Bee specimen record database. https://www.discoverlife.org/mp/20l?id=AMNH_BEES108796

16 American Museum of Natural History, Bee specimen record database. https://www.discoverlife.org/mp/20l?id=AMNH_BEES108793

17 American Museum of Natural History, Bee specimen record database. https://www.discoverlife.org/mp/20l?id=AMNH_BEES108806

18 American Museum of Natural History, Bee specimen record database. https://www.discoverlife.org/mp/20l?id=AMNH_BEES108797

19 American Museum of Natural History, Bee specimen record database. https://www.discoverlife.org/mp/20l?id=AMNH_BEES108798

20 American Museum of Natural History, Bee specimen record database. https://www.discoverlife.org/mp/20l?id=AMNH_BEES108772

21 American Museum of Natural History, Bee specimen record database. https://www.discoverlife.org/mp/20l?id=AMNH_BEES108769

22 American Museum of Natural History, Bee specimen record database. https://www.discoverlife.org/mp/20l?id=AMNH_BEES108771

23 American Museum of Natural History, Bee specimen record database. https://www.discoverlife.org/mp/20l?id=AMNH_BEES108767

24 iNaturalist.org: iNaturalist research-grade observations https://www.discoverlife.org/mp/20l?id=INAT32613351

25 American Museum of Natural History, Bee specimen record database. https://www.discoverlife.org/mp/20l?id=AMNH_BEES108768

26 American Museum of Natural History, Bee specimen record database. https://www.discoverlife.org/mp/20l?id=AMNH_BEES108742

27 American Museum of Natural History, Bee specimen record database. https://www.discoverlife.org/mp/20l?id=AMNH_BEES108750

28 American Museum of Natural History, Bee specimen record database. https://www.discoverlife.org/mp/20l?id=AMNH_BEES108743

29		American Museum of Natural History, Bee specimen record database. https://www.discoverlife.org/mp/20l?id=AMNH_BEES108745
30		York University CCDB-01563 G10 https://www.discoverlife.org/mp/20l?id=BOLD__BOWGF367_09
31		American Museum of Natural History, Bee specimen record database. https://www.discoverlife.org/mp/20l?id=AMNH_BEES108765
32		American Museum of Natural History, Bee specimen record database. https://www.discoverlife.org/mp/20l?id=AMNH_BEES108740
33		American Museum of Natural History, Bee specimen record database. https://www.discoverlife.org/mp/20l?id=AMNH_BEES108756
34		American Museum of Natural History, Bee specimen record database. https://www.discoverlife.org/mp/20l?id=AMNH_BEES108759
35		American Museum of Natural History, Bee specimen record database. https://www.discoverlife.org/mp/20l?id=AMNH_BEES108753
36		American Museum of Natural History, Bee specimen record database. https://www.discoverlife.org/mp/20l?id=AMNH_BEES108754
37		American Museum of Natural History, Bee specimen record database. https://www.discoverlife.org/mp/20l?id=AMNH_BEES108760
38		American Museum of Natural History, Bee specimen record database. https://www.discoverlife.org/mp/20l?id=AMNH_BEES108757
39	*Ceratina smaragdula*	American Museum of Natural History, Bee specimen record database. https://www.discoverlife.org/mp/20l?id=AMNH_BEE00031688
40		American Museum of Natural History, Bee specimen record database. https://www.discoverlife.org/mp/20l?id=AMNH_BEES109701<br>American Museum of Natural History, Bee specimen record database. https://www.discoverlife.org/mp/20l?id=AMNH_BEE00031686<br>USDA-ARS Bee Biology and Systematics Laboratory: Bee Biology and Systematics Laboratory https://www.discoverlife.org/mp/20l?id=BBSL347461<br>USDA-ARS Bee Biology and Systematics Laboratory: Bee Biology and Systematics Laboratory https://www.discoverlife.org/mp/20l?id=BBSL347612<br>American Museum of Natural History, Bee specimen record database https://www.discoverlife.org/mp/20l?id=AMNH_BEES109704<br>American Museum of Natural History, Bee specimen record database https://www.discoverlife.org/mp/20l?id=AMNH_BEES109770<br>American Museum of Natural History, Bee specimen record database https://www.discoverlife.org/mp/20l?id=AMNH_BEES109769<br>American Museum of Natural History, Bee specimen record database https://www.discoverlife.org/mp/20l?id=AMNH_BEES109772<br>American Museum of Natural History, Bee specimen record database https://www.discoverlife.org/mp/20l?id=AMNH_BEES109765<br>American Museum of Natural History, Bee specimen record database https://www.discoverlife.org/mp/20l?id=AMNH_BEE00031722<br>American Museum of Natural History, Bee specimen record database https://www.discoverlife.org/mp/20l?id=AMNH_BEES109764

American Museum of Natural History, Bee specimen record database
iNaturalist.org: iNaturalist research-grade observations
https://www.discoverlife.org/mp/20l?id=INAT13825260
iNaturalist.org: iNaturalist research-grade observations
https://www.discoverlife.org/mp/20l?id=INAT8483618
American Museum of Natural History, Bee specimen record database
https://www.discoverlife.org/mp/20l?id=AMNH_BEES109767
iNaturalist.org: iNaturalist research-grade observations
https://www.discoverlife.org/mp/20l?id=INAT13865626
American Museum of Natural History, Bee specimen record database
https://www.discoverlife.org/mp/20l?id=AMNH_BEES109771
American Museum of Natural History, Bee specimen record database
https://www.discoverlife.org/mp/20l?id=AMNH_BEES109729
American Museum of Natural History, Bee specimen record database
https://www.discoverlife.org/mp/20l?id=AMNH_BEES109728
American Museum of Natural History, Bee specimen record database
https://www.discoverlife.org/mp/20l?id=AMNH_BEES109722
American Museum of Natural History, Bee specimen record database
https://www.discoverlife.org/mp/20l?id=AMNH_BEES109763
American Museum of Natural History, Bee specimen record database
https://www.discoverlife.org/mp/20l?id=AMNH_BEES109720
York University
https://www.discoverlife.org/mp/20l?id=BOLD__BOWGF359_09
American Museum of Natural History, Bee specimen record database
https://www.discoverlife.org/mp/20l?id=AMNH_BEES109726
American Museum of Natural History, Bee specimen record database
https://www.discoverlife.org/mp/20l?id=AMNH_BEES109761
American Museum of Natural History, Bee specimen record database
https://www.discoverlife.org/mp/20l?id=AMNH_BEES109760
American Museum of Natural History, Bee specimen record database
https://www.discoverlife.org/mp/20l?id=AMNH_BEES109752
American Museum of Natural History, Bee specimen record database
https://www.discoverlife.org/mp/20l?id=AMNH_BEES109746
American Museum of Natural History, Bee specimen record database
https://www.discoverlife.org/mp/20l?id=AMNH_BEES109738
American Museum of Natural History, Bee specimen record database
iNaturalist.org: iNaturalist research-grade observations
https://www.discoverlife.org/mp/20l?id=INAT32327326
American Museum of Natural History, Bee specimen record database
https://www.discoverlife.org/mp/20l?id=AMNH_BEES109750
American Museum of Natural History, Bee specimen record database
https://www.discoverlife.org/mp/20l?id=AMNH_BEES109751
American Museum of Natural History, Bee specimen record database
https://www.discoverlife.org/mp/20l?id=AMNH_BEES109709
American Museum of Natural History, Bee specimen record database
https://www.discoverlife.org/mp/20l?id=AMNH_BEES109710

	American Museum of Natural History, Bee specimen record database https://www.discoverlife.org/mp/20l?id=AMNH_BEES109711
	American Museum of Natural History, Bee specimen record database https://www.discoverlife.org/mp/20l?id=AMNH_BEES109793
	iNaturalist.org: iNaturalist research-grade observations https://www.discoverlife.org/mp/20l?id=INAT9751564
	American Museum of Natural History, Bee specimen record database https://www.discoverlife.org/mp/20l?id=AMNH_BEES109790
	American Museum of Natural History, Bee specimen record database https://www.discoverlife.org/mp/20l?id=AMNH_BEES109716
	American Museum of Natural History, Bee specimen record database https://www.discoverlife.org/mp/20l?id=AMNH_BEES109714
	American Museum of Natural History, Bee specimen record database https://www.discoverlife.org/mp/20l?id=AMNH_BEES109707
	American Museum of Natural History, Bee specimen record database https://www.discoverlife.org/mp/20l?id=AMNH_BEES109706
Lasioglossum impavidum	USDA-ARS Bee Biology and Systematics Laboratory: Bee Biology and Systematics Laboratory https://www.discoverlife.org/mp/20l?id=BBSL435935
	USDA-ARS Bee Biology and Systematics Laboratory: Bee Biology and Systematics Laboratory https://www.discoverlife.org/mp/20l?id=BBSL454632
	USDA-ARS Bee Biology and Systematics Laboratory: Bee Biology and Systematics Laboratory https://www.discoverlife.org/mp/20l?id=BBSL350436
	USDA-ARS Bee Biology and Systematics Laboratory: Bee Biology and Systematics Laboratory https://www.discoverlife.org/mp/20l?id=BBSL510282
	USDA-ARS Bee Biology and Systematics Laboratory: Bee Biology and Systematics Laboratory https://www.discoverlife.org/mp/20l?id=BBSL451103
	USDA-ARS Bee Biology and Systematics Laboratory: Bee Biology and Systematics Laboratory https://www.discoverlife.org/mp/20l?id=BBSL456477
	USDA-ARS Bee Biology and Systematics Laboratory: Bee Biology and Systematics Laboratory https://www.discoverlife.org/mp/20l?id=BBSL__ZION31023
	USDA-ARS Bee Biology and Systematics Laboratory: Bee Biology and Systematics Laboratory https://www.discoverlife.org/mp/20l?id=BBSL509896
	USDA-ARS Bee Biology and Systematics Laboratory: Bee Biology and Systematics Laboratory https://www.discoverlife.org/mp/20l?id=BBSL321662
	USDA-ARS Bee Biology and Systematics Laboratory: Bee Biology and Systematics Laboratory https://www.discoverlife.org/mp/20l?id=BBSL509899

	USDA-ARS Bee Biology and Systematics Laboratory: Bee Biology and Systematics Laboratory https://www.discoverlife.org/mp/20l?id=BBSL509906 USDA-ARS Bee Biology and Systematics Laboratory: Bee Biology and Systematics Laboratory https://www.discoverlife.org/mp/20l?id=BBSL262958 USDA-ARS Bee Biology and Systematics Laboratory: Bee Biology and Systematics Laboratory https://www.discoverlife.org/mp/20l?id=BBSL301324 USDA-ARS Bee Biology and Systematics Laboratory: Bee Biology and Systematics Laboratory https://www.discoverlife.org/mp/20l?id=BBSL300071 USDA-ARS Bee Biology and Systematics Laboratory: Bee Biology and Systematics Laboratory https://www.discoverlife.org/mp/20l?id=BBSL509903 USDA-ARS Bee Biology and Systematics Laboratory: Bee Biology and Systematics Laboratory https://www.discoverlife.org/mp/20l?id=BBSL279758 USDA-ARS Bee Biology and Systematics Laboratory: Bee Biology and Systematics Laboratory https://www.discoverlife.org/mp/20l?id=BBSL93812 USDA-ARS Bee Biology and Systematics Laboratory: Bee Biology and Systematics Laboratory https://www.discoverlife.org/mp/20l?id=BBSL313284
Lasioglossum microlepoides	USGS. Sam Droege https://www.discoverlife.org/mp/20l?id=USGS_DRO292436 USGS. Sam Droege https://www.discoverlife.org/mp/20l?id=USGS_DRO404813 USDA-ARS Bee Biology and Systematics Laboratory: Bee Biology and Systematics Laboratory https://www.discoverlife.org/mp/20l?id=BBSL583997 USDA-ARS Bee Biology and Systematics Laboratory: Bee Biology and Systematics Laboratory https://www.discoverlife.org/mp/20l?id=BBSL583997 USDA-ARS Bee Biology and Systematics Laboratory: Bee Biology and Systematics Laboratory https://www.discoverlife.org/mp/20l?id=BBSL339028 USDA-ARS Bee Biology and Systematics Laboratory: Bee Biology and Systematics Laboratory https://www.discoverlife.org/mp/20l?id=BBSL297760 USDA-ARS Bee Biology and Systematics Laboratory: Bee Biology and Systematics Laboratory https://www.discoverlife.org/mp/20l?id=BBSL94268 USDA-ARS Bee Biology and Systematics Laboratory: Bee Biology and Systematics Laboratory https://www.discoverlife.org/mp/20l?id=BBSL93867

iNaturalist.org: iNaturalist research-grade observations
https://www.discoverlife.org/mp/20l?id=INAT2769134
iNaturalist.org: iNaturalist research-grade observations
https://www.discoverlife.org/mp/20l?id=INAT3459005
University of California Riverside Entomoglogy Museum database
https://www.discoverlife.org/mp/20l?id=UCREM510
USDA-ARS Bee Biology and Systematics Laboratory: Bee Biology and Systematics Laboratory
https://www.discoverlife.org/mp/20l?id=BBSL323275
USDA-ARS Bee Biology and Systematics Laboratory: Bee Biology and Systematics Laboratory
https://www.discoverlife.org/mp/20l?id=BBSL322921
USDA-ARS Bee Biology and Systematics Laboratory: Bee Biology and Systematics Laboratory
https://www.discoverlife.org/mp/20l?id=BBSL281346
USDA-ARS Bee Biology and Systematics Laboratory: Bee Biology and Systematics Laboratory
https://www.discoverlife.org/mp/20l?id=BBSL509975
USDA-ARS Bee Biology and Systematics Laboratory: Bee Biology and Systematics Laboratory
https://www.discoverlife.org/mp/20l?id=BBSL93917
USDA-ARS Bee Biology and Systematics Laboratory: Bee Biology and Systematics Laboratory
https://www.discoverlife.org/mp/20l?id=BBSL278593
USDA-ARS Bee Biology and Systematics Laboratory: Bee Biology and Systematics Laboratory
https://www.discoverlife.org/mp/20l?id=BBSL509972
USDA-ARS Bee Biology and Systematics Laboratory: Bee Biology and Systematics Laboratory
https://www.discoverlife.org/mp/20l?id=BBSL262697
USDA-ARS Bee Biology and Systematics Laboratory: Bee Biology and Systematics Laboratory
https://www.discoverlife.org/mp/20l?id=BBSL299424
USDA-ARS Bee Biology and Systematics Laboratory: Bee Biology and Systematics Laboratory
https://www.discoverlife.org/mp/20l?id=BBSL319435
USDA-ARS Bee Biology and Systematics Laboratory: Bee Biology and Systematics Laboratory
https://www.discoverlife.org/mp/20l?id=BBSL319366
York University. BOLD database.
https://www.discoverlife.org/mp/20l?id=BOLD__DLII1000_07
USGS. Sam Droege
https://www.discoverlife.org/mp/20l?id=USGS_DRO332280
American Museum of Natural History. Bee Specimen Record database.
https://www.discoverlife.org/mp/20l?id=AMNH_BEE00119936

American Museum of Natural History. Bee Specimen Record database.
https://www.discoverlife.org/mp/20l?id=AMNH_BEE00119939
USDA-ARS Bee Biology and Systematics Laboratory: Bee Biology and Systematics Laboratory
https://www.discoverlife.org/mp/20l?id=BBSL321175
USDA-ARS Bee Biology and Systematics Laboratory: Bee Biology and Systematics Laboratory
https://www.discoverlife.org/mp/20l?id=BBSL321006
USDA-ARS Bee Biology and Systematics Laboratory: Bee Biology and Systematics Laboratory
https://www.discoverlife.org/mp/20l?id=BBSL324994
USDA-ARS Bee Biology and Systematics Laboratory: Bee Biology and Systematics Laboratory
https://www.discoverlife.org/mp/20l?id=BBSL135422
USDA-ARS Bee Biology and Systematics Laboratory: Bee Biology and Systematics Laboratory
https://www.discoverlife.org/mp/20l?id=BBSL__ZION32709
USDA-ARS Bee Biology and Systematics Laboratory: Bee Biology and Systematics Laboratory
https://www.discoverlife.org/mp/20l?id=BBSL375168
USDA-ARS Bee Biology and Systematics Laboratory: Bee Biology and Systematics Laboratory
https://www.discoverlife.org/mp/20l?id=BBSL492017
USDA-ARS Bee Biology and Systematics Laboratory: Bee Biology and Systematics Laboratory
https://www.discoverlife.org/mp/20l?id=BBSL33972
USGS. Sam Droege
https://www.discoverlife.org/mp/20l?id=USGS_DRO219945
USGS. Sam Droege
https://www.discoverlife.org/mp/20l?id=USGS_DRO217300
USGS. Sam Droege
https://www.discoverlife.org/mp/20l?id=USGS_DRO175083
USDA-ARS Bee Biology and Systematics Laboratory: Bee Biology and Systematics Laboratory
https://www.discoverlife.org/mp/20l?id=BBSL33971
USDA-ARS Bee Biology and Systematics Laboratory: Bee Biology and Systematics Laboratory
https://www.discoverlife.org/mp/20l?id=BBSL664493
USDA-ARS Bee Biology and Systematics Laboratory: Bee Biology and Systematics Laboratory
https://www.discoverlife.org/mp/20l?id=BBSL109934
USDA-ARS Bee Biology and Systematics Laboratory: Bee Biology and Systematics Laboratory
https://www.discoverlife.org/mp/20l?id=BBSL750705

	American Museum of Natural History. Bee Specimen Record database.<br>https://www.discoverlife.org/mp/20l?id=AMNH_BEE00119940<br>American Museum of Natural History. Bee Specimen Record database.<br>https://www.discoverlife.org/mp/20l?id=AMNH_BEE00208178<br>USDA-ARS Bee Biology and Systematics Laboratory: Bee Biology and Systematics Laboratory<br>https://www.discoverlife.org/mp/20l?id=BBSL109928<br>USGS. Sam Droege<br>https://www.discoverlife.org/mp/20l?id=USGS_DRO225830<br>USDA-ARS Bee Biology and Systematics Laboratory: Bee Biology and Systematics Laboratory<br>https://www.discoverlife.org/mp/20l?id=BBSL750569<br>Kansas Natural History Museum Snow Entomology Collection database. https://www.discoverlife.org/mp/20l?id=KSEM313640<br>Kansas Natural History Museum Snow Entomology Collection database. https://www.discoverlife.org/mp/20l?id=KSEM313645
Lasioglossum puteulanum	USGS. Sam Droege<br>https://www.discoverlife.org/mp/20l?id=USGS_DRO141676<br>USGS. Sam Droege<br>https://www.discoverlife.org/mp/20l?id=USGS_DRO266346<br>USGS. Sam Droege<br>https://www.discoverlife.org/mp/20l?id=USGS_DRO208732<br>USGS. Sam Droege<br>https://www.discoverlife.org/mp/20l?id=USGS_DRO228014<br>USGS. Sam Droege<br>https://www.discoverlife.org/mp/20l?id=USGS_DRO614555<br>USGS. Sam Droege<br>https://www.discoverlife.org/mp/20l?id=USGS_DRO185679<br>USGS. Sam Droege<br>https://www.discoverlife.org/mp/20l?id=USGS_DRO208355<br>USGS. Sam Droege<br>https://www.discoverlife.org/mp/20l?id=USGS_DRO208653<br>USGS. Sam Droege<br>https://www.discoverlife.org/mp/20l?id=USGS_DRO211657<br>USGS. Sam Droege<br>https://www.discoverlife.org/mp/20l?id=USGS_DRO208511<br>USGS. Sam Droege<br>https://www.discoverlife.org/mp/20l?id=USGS_DRO218272<br>York University Bold database.<br>https://www.discoverlife.org/mp/20l?id=BOLD__DIAL487_06<br>USGS. Sam Droege<br>https://www.discoverlife.org/mp/20l?id=USGS_DRO119394<br>USGS. Sam Droege<br>https://www.discoverlife.org/mp/20l?id=USGS_DRO125971

USGS. Sam Droege
https://www.discoverlife.org/mp/20l?id=USGS_DRO124162
USGS. Sam Droege
https://www.discoverlife.org/mp/20l?id=USGS_DRO125682
USGS. Sam Droege
https://www.discoverlife.org/mp/20l?id=USGS_DRO125583
USGS. Sam Droege
https://www.discoverlife.org/mp/20l?id=USGS_DRO125913
USGS. Sam Droege
https://www.discoverlife.org/mp/20l?id=USGS_DRO119426
USGS. Sam Droege
https://www.discoverlife.org/mp/20l?id=USGS_DRO228014
USGS. Sam Droege
https://www.discoverlife.org/mp/20l?id=USGS_DRO614555
York University Bold database
https://www.discoverlife.org/mp/20l?id=BOLD__DLII465_07
York University Bold database
https://www.discoverlife.org/mp/20l?id=BOLD__DIAL498_06
USGS. Sam Droege
https://www.discoverlife.org/mp/20l?id=USGS_DRO185679
USGS. Sam Droege
https://www.discoverlife.org/mp/20l?id=USGS_DRO485447
USGS. Sam Droege
https://www.discoverlife.org/mp/20l?id=USGS_DRO419180
USGS. Sam Droege
https://www.discoverlife.org/mp/20l?id=USGS_DRO322750
USGS. Sam Droege
https://www.discoverlife.org/mp/20l?id=USGS_DRO322627
USGS. Sam Droege
https://www.discoverlife.org/mp/20l?id=USGS_DRO322772
USGS. Sam Droege
https://www.discoverlife.org/mp/20l?id=USGS_DRO456140
USGS. Sam Droege
https://www.discoverlife.org/mp/20l?id=USGS_DRO032314
York University BOLD database.
https://www.discoverlife.org/mp/20l?id=BOLD__DIAL029_06
York University BOLD database.
https://www.discoverlife.org/mp/20l?id=BOLD__DLII472_07
York University BOLD database
https://www.discoverlife.org/mp/20l?id=BOLD__DIAL514_06
York University BOLD database
https://www.discoverlife.org/mp/20l?id=BOLD__DIAL514_06
American Museum of Natural History, Bee Specimen Record database
https://www.discoverlife.org/mp/20l?id=AMNH_BEE00210751

	American Museum of Natural History, Bee Specimen Record database<br>https://www.discoverlife.org/mp/20l?id=AMNH_BEE00116083<br>USGS. Sam Droege<br>https://www.discoverlife.org/mp/20l?id=USGS_DRO093456<br>USGS. Sam Droege<br>https://www.discoverlife.org/mp/20l?id=USGS_DRO456115<br>USGS. Sam Droege<br>https://www.discoverlife.org/mp/20l?id=USGS_DRO344954<br>USGS. Sam Droege<br>https://www.discoverlife.org/mp/20l?id=USGS_DRO121910<br>American Museum of Natural History, Bee Specimen Record database<br>https://www.discoverlife.org/mp/20l?id=AMNH_BEE00262005<br>American Museum of Natural History, Bee Specimen Record database<br>https://www.discoverlife.org/mp/20l?id=AMNH_BEE00076617<br>American Museum of Natural History, Bee Specimen Record database<br>https://www.discoverlife.org/mp/20l?id=AMNH_BEE00076614
Megachile umbripennis	American Museum of Natural History, Bee Specimen Record database<br>https://www.discoverlife.org/mp/20l?id=AMNH_BEE00251347<br>USGS. Sam Droege<br>https://www.discoverlife.org/mp/20l?id=USGS_DRO375353<br>American Museum of Natural History, Bee Specimen Record database<br>https://www.discoverlife.org/mp/20l?id=AMNH_BEES105501<br>American Museum of Natural History, Bee Specimen Record database<br>https://www.discoverlife.org/mp/20l?id=AMNH_BEES105502<br>American Museum of Natural History, Bee Specimen Record database<br>https://www.discoverlife.org/mp/20l?id=AMNH_BEES105503<br>American Museum of Natural History, Bee Specimen Record database<br>https://www.discoverlife.org/mp/20l?id=AMNH_BEES105504<br>American Museum of Natural History, Bee Specimen Record database<br>https://www.discoverlife.org/mp/20l?id=AMNH_BEES105527<br>American Museum of Natural History, Bee Specimen Record database<br>https://www.discoverlife.org/mp/20l?id=AMNH_BEES105538<br>American Museum of Natural History, Bee Specimen Record database<br>https://www.discoverlife.org/mp/20l?id=AMNH_BEES105537

American Museum of Natural History, Bee Specimen Record database
https://www.discoverlife.org/mp/20l?id=AMNH_BEES105525
American Museum of Natural History, Bee Specimen Record database
https://www.discoverlife.org/mp/20l?id=AMNH_BEES105535
American Museum of Natural History, Bee Specimen Record database
https://www.discoverlife.org/mp/20l?id=AMNH_BEES105529
American Museum of Natural History, Bee Specimen Record database
https://www.discoverlife.org/mp/20l?id=AMNH_BEES105533
American Museum of Natural History, Bee Specimen Record database
https://www.discoverlife.org/mp/20l?id=AMNH_BEES105536
American Museum of Natural History, Bee Specimen Record database
https://www.discoverlife.org/mp/20l?id=AMNH_BEES105532
American Museum of Natural History, Bee Specimen Record database
https://www.discoverlife.org/mp/20l?id=AMNH_BEES105532
USDA-ARS Bee Biology and Systematics Laboratory: Bee Biology and Systematics Laboratory
https://www.discoverlife.org/mp/20l?id=BBSL746023
iNaturalist.org: iNaturalist research-grade observations
https://www.discoverlife.org/mp/20l?id=INAT34159126
iNaturalist.org: iNaturalist research-grade observations
https://www.discoverlife.org/mp/20l?id=INAT34622353
iNaturalist.org: iNaturalist research-grade observations
https://www.discoverlife.org/mp/20l?id=INAT12373299
iNaturalist.org: iNaturalist research-grade observations
https://www.discoverlife.org/mp/20l?id=INAT16860468
iNaturalist.org: iNaturalist research-grade observations
https://www.discoverlife.org/mp/20l?id=INAT19688947
American Museum of Natural History, Bee Specimen Record database
https://www.discoverlife.org/mp/20l?id=AMNH_BEES105526
American Museum of Natural History, Bee Specimen Record database
https://www.discoverlife.org/mp/20l?id=AMNH_BEES105531
American Museum of Natural History, Bee Specimen Record database
https://www.discoverlife.org/mp/20l?id=AMNH_BEES105511
American Museum of Natural History, Bee Specimen Record database
https://www.discoverlife.org/mp/20l?id=AMNH_BEES105509

		American Museum of Natural History, Bee Specimen Record database https://www.discoverlife.org/mp/20l?id=AMNH_BEES105510<br>American Museum of Natural History, Bee Specimen Record database https://www.discoverlife.org/mp/20l?id=AMNH_BEES105508<br>American Museum of Natural History, Bee Specimen Record database https://www.discoverlife.org/mp/20l?id=AMNH_BEES105521<br>American Museum of Natural History, Bee Specimen Record database https://www.discoverlife.org/mp/20l?id=AMNH_BEES105514<br>American Museum of Natural History, Bee Specimen Record database https://www.discoverlife.org/mp/20l?id=AMNH_BEES105522<br>American Museum of Natural History, Bee Specimen Record database https://www.discoverlife.org/mp/20l?id=AMNH_BEES105513<br>American Museum of Natural History, Bee Specimen Record database https://www.discoverlife.org/mp/20l?id=AMNH_BEE00010198<br>American Museum of Natural History, Bee Specimen Record database https://www.discoverlife.org/mp/20l?id=AMNH_BEE00010199<br>iNaturalist.org: iNaturalist research-grade observations https://www.discoverlife.org/mp/20l?id=INAT12476358<br>USDA-ARS Bee Biology and Systematics Laboratory: Bee Biology and Systematics Laboratory https://www.discoverlife.org/mp/20l?id=BBSL743787
	Xylocopa sonorina	iNaturalist.org: iNaturalist research-grade observations https://www.discoverlife.org/mp/20l?id=INAT29692584<br>University of California, Riverside, Entomology Research Museum database https://www.discoverlife.org/mp/20l?id=UCRC_ENT60071<br>American Museum of Natural History, Bee Specimen Record database https://www.discoverlife.org/mp/20l?id=AMNH_BEES23931<br>University of California, Riverside, Entomology Research Museum database https://www.discoverlife.org/mp/20l?id=UCRC_ENT144059<br>University of California, Riverside, Entomology Research Museum database https://www.discoverlife.org/mp/20l?id=UCRC_ENT42827<br>University of California, Riverside, Entomology Research Museum database https://www.discoverlife.org/mp/20l?id=UCRC_ENT331342

(b) Non-native (Hawai‘i) records.

ID	Species	Institution

1	*Ceratina dentipes*	iNaturalist.org: iNaturalist research-grade observations https://www.discoverlife.org/mp/20l?id=INAT4449583
2	*Ceratina smaragdula*	iNaturalist.org: iNaturalist research-grade observations https://www.discoverlife.org/mp/20l?id=INAT17525366
3		USGS. Sam Droege https://www.discoverlife.org/mp/20l?id=USGS_DRO395295
4		USGS. Sam Droege https://www.discoverlife.org/mp/20l?id=USGS_DRO296883
5		USGS. Sam Droege https://www.discoverlife.org/mp/20l?id=USGS_DRO296801
6		USGS. Sam Droege https://www.discoverlife.org/mp/20l?id=USGS_DRO297058
7		iNaturalist.org: iNaturalist research-grade observations https://www.discoverlife.org/mp/20l?id=INAT18875352
8		USGS. Sam Droege https://www.discoverlife.org/mp/20l?id=USGS_DRO299800
9		USGS. Sam Droege https://www.discoverlife.org/mp/20l?id=USGS_DRO297262
10		iNaturalist.org: iNaturalist research-grade observations https://www.discoverlife.org/mp/20l?id=INAT14505178
11		USGS. Sam Droege https://www.discoverlife.org/mp/20l?id=USGS_DRO373639
12		USGS. Sam Droege https://www.discoverlife.org/mp/20l?id=USGS_DRO373652
13		USGS. Sam Droege https://www.discoverlife.org/mp/20l?id=USGS_DRO375096
14		USGS. Sam Droege https://www.discoverlife.org/mp/20l?id=USGS_DRO376362
15		USGS. Sam Droege https://www.discoverlife.org/mp/20l?id=USGS_DRO375005
16		USGS. Sam Droege https://www.discoverlife.org/mp/20l?id=USGS_DRO376308
17		USGS. Sam Droege https://www.discoverlife.org/mp/20l?id=USGS_DRO374902
18		USGS. Sam Droege https://www.discoverlife.org/mp/20l?id=USGS_DRO374902
19		USGS. Sam Droege https://www.discoverlife.org/mp/20l?id=USGS_DRO333603
20		USGS. Sam Droege https://www.discoverlife.org/mp/20l?id=USGS_DRO374964
		USGS. Sam Droege https://www.discoverlife.org/mp/20l?id=USGS_DRO374158
		iNaturalist.org: iNaturalist research-grade observations https://www.discoverlife.org/mp/20l?id=INAT18533202
		iNaturalist.org: iNaturalist research-grade observations https://www.discoverlife.org/mp/20l?id=INAT5052542

	iNaturalist.org: iNaturalist research-grade observations https://www.discoverlife.org/mp/20l?id=INAT28843815
Lasioglossum impavidum	USGS. Sam Droege https://www.discoverlife.org/mp/20l?id=USGS_DRO374087 USGS. Sam Droege https://www.discoverlife.org/mp/20l?id=USGS_DRO333606 USGS. Sam Droege https://www.discoverlife.org/mp/20l?id=USGS_DRO374961 USGS. Sam Droege iNaturalist.org: iNaturalist research-grade observations https://www.discoverlife.org/mp/20l?id=INAT17437721
Lasioglossum microlepoides	USGS. Sam Droege https://www.discoverlife.org/mp/20l?id=USGS_DRO296796 USGS. Sam Droege https://www.discoverlife.org/mp/20l?id=USGS_DRO296855 USGS. Sam Droege https://www.discoverlife.org/mp/20l?id=USGS_DRO297052 USGS. Sam Droege https://www.discoverlife.org/mp/20l?id=USGS_DRO301521 USGS. Sam Droege https://www.discoverlife.org/mp/20l?id=USGS_DRO301641 USGS. Sam Droege https://www.discoverlife.org/mp/20l?id=USGS_DRO296895 USGS. Sam Droege https://www.discoverlife.org/mp/20l?id=USGS_DRO300004 USGS. Sam Droege https://www.discoverlife.org/mp/20l?id=USGS_DRO297264 USGS. Sam Droege https://www.discoverlife.org/mp/20l?id=USGS_DRO300195 USGS. Sam Droege https://www.discoverlife.org/mp/20l?id=USGS_DRO299974
Lasioglossum puteulanum	None
Megachile umbripennis	iNaturalist.org: iNaturalist research-grade observations https://www.discoverlife.org/mp/20l?id=INAT17499827 University of California Riverside Entomology Resaerch Museum database https://www.discoverlife.org/mp/20l?id=UCRC_ENT231547 iNaturalist.org: iNaturalist research-grade observations https://www.discoverlife.org/mp/20l?id=INAT18512497 USDA-ARS Bee Biology and Systematics Laboratory: Bee Biology and Systematics Laboratory https://www.discoverlife.org/mp/20l?id=BBSL743780 USDA-ARS Bee Biology and Systematics Laboratory: Bee Biology and Systematics Laboratory https://www.discoverlife.org/mp/20l?id=BBSL743783

	American Museum of Natural History, Bee Specimen Record database https://www.discoverlife.org/mp/20l?id=AMNH_BEE00010195
	USGS. Sam Droege https://www.discoverlife.org/mp/20l?id=USGS_DRO376328
	USGS. Sam Droege https://www.discoverlife.org/mp/20l?id=USGS_DRO376359
	USGS. Sam Droege https://www.discoverlife.org/mp/20l?id=USGS_DRO375011
	USGS. Sam Droege https://www.discoverlife.org/mp/20l?id=USGS_DRO374989
	USGS. Sam Droege https://www.discoverlife.org/mp/20l?id=USGS_DRO375060
	USGS. Sam Droege https://www.discoverlife.org/mp/20l?id=USGS_DRO374907
	USGS. Sam Droege https://www.discoverlife.org/mp/20l?id=USGS_DRO376739
	USGS. Sam Droege https://www.discoverlife.org/mp/20l?id=USGS_DRO374085
	USGS. Sam Droege https://www.discoverlife.org/mp/20l?id=USGS_DRO374136
	USGS. Sam Droege https://www.discoverlife.org/mp/20l?id=USGS_DRO374171
	USGS. Sam Droege https://www.discoverlife.org/mp/20l?id=USGS_DRO376137
	iNaturalist.org: iNaturalist research-grade observations https://www.discoverlife.org/mp/20l?id=INAT11189190
	University of Connecticut Insect Collection database https://www.discoverlife.org/mp/20l?id=UCMS_ENT00040949
	University of Connecticut Insect Collection database https://www.discoverlife.org/mp/20l?id=UCMS_ENT00040950
	University of California Riverside Entomology Resaerch Museum database https://www.discoverlife.org/mp/20l?id=UCRC_ENT231561
Xylocopa sonorina	iNaturalist.org: iNaturalist research-grade observations https://www.discoverlife.org/mp/20l?id=INAT20986503
	iNaturalist.org: iNaturalist research-grade observations https://www.discoverlife.org/mp/20l?id=INAT32998580
	iNaturalist.org: iNaturalist research-grade observations https://www.discoverlife.org/mp/20l?id=INAT19833559
	iNaturalist.org: iNaturalist research-grade observations https://www.discoverlife.org/mp/20l?id=INAT10056255
	iNaturalist.org: iNaturalist research-grade observations https://www.discoverlife.org/mp/20l?id=INAT2504994
	iNaturalist.org: iNaturalist research-grade observations https://www.discoverlife.org/mp/20l?id=INAT2774328

iNaturalist.org: iNaturalist research-grade observations
https://www.discoverlife.org/mp/20l?id=INAT655652
iNaturalist.org: iNaturalist research-grade observations
https://www.discoverlife.org/mp/20l?id=INAT4816518
American Museum of Natural History, Bee Specimen Record database
https://www.discoverlife.org/mp/20l?id=AMNH_BEE00250591
American Museum of Natural History, Bee Specimen Record database
https://www.discoverlife.org/mp/20l?id=AMNH_BEE00250589
USGS Sam Droege
https://www.discoverlife.org/mp/20l?id=USGS_DRO297062
USGS Sam Droege
https://www.discoverlife.org/mp/20l?id=USGS_DRO296805
USGS Sam Droege
https://www.discoverlife.org/mp/20l?id=USGS_DRO301589
USGS Sam Droege
https://www.discoverlife.org/mp/20l?id=USGS_DRO299585
USGS Sam Droege
https://www.discoverlife.org/mp/20l?id=USGS_DRO297346
USGS Sam Droege
https://www.discoverlife.org/mp/20l?id=USGS_DRO296803
USGS Sam Droege
https://www.discoverlife.org/mp/20l?id=USGS_DRO375002
iNaturalist.org: iNaturalist research-grade observations
https://www.discoverlife.org/mp/20l?id=INAT35101898
iNaturalist.org: iNaturalist research-grade observations
https://www.discoverlife.org/mp/20l?id=INAT17555547
iNaturalist.org: iNaturalist research-grade observations
https://www.discoverlife.org/mp/20l?id=INAT10266972
iNaturalist.org: iNaturalist research-grade observations
https://www.discoverlife.org/mp/20l?id=INAT9526730
iNaturalist.org: iNaturalist research-grade observations
https://www.discoverlife.org/mp/20l?id=INAT4052346
iNaturalist.org: iNaturalist research-grade observations
https://www.discoverlife.org/mp/20l?id=INAT18460274
iNaturalist.org: iNaturalist research-grade observations
https://www.discoverlife.org/mp/20l?id=INAT2777729
iNaturalist.org: iNaturalist research-grade observations
https://www.discoverlife.org/mp/20l?id=INAT3991007
USGS Sam Droege
https://www.discoverlife.org/mp/20l?id=USGS_DRO376370
USGS Sam Droege
https://www.discoverlife.org/mp/20l?id=USGS_DRO376754
USGS Sam Droege
https://www.discoverlife.org/mp/20l?id=USGS_DRO374165

USGS Sam Droege
https://www.discoverlife.org/mp/20l?id=USGS_DRO376740
USGS Sam Droege
https://www.discoverlife.org/mp/20l?id=USGS_DRO376120
USGS Sam Droege
https://www.discoverlife.org/mp/20l?id=USGS_DRO376185
USGS Sam Droege
https://www.discoverlife.org/mp/20l?id=USGS_DRO376389
USGS Sam Droege
https://www.discoverlife.org/mp/20l?id=USGS_DRO376388
iNaturalist.org: iNaturalist research-grade observations
https://www.discoverlife.org/mp/20l?id=INAT35157196
iNaturalist.org: iNaturalist research-grade observations
https://www.discoverlife.org/mp/20l?id=INAT262095
iNaturalist.org: iNaturalist research-grade observations
https://www.discoverlife.org/mp/20l?id=INAT18147742
iNaturalist.org: iNaturalist research-grade observations
https://www.discoverlife.org/mp/20l?id=INAT4438293
iNaturalist.org: iNaturalist research-grade observations
https://www.discoverlife.org/mp/20l?id=INAT5054349
iNaturalist.org: iNaturalist research-grade observations
https://www.discoverlife.org/mp/20l?id=INAT28845441
iNaturalist.org: iNaturalist research-grade observations
https://www.discoverlife.org/mp/20l?id=INAT4245327
USDA-ARS Bee Biology and Systematics Laboratory: Bee Biology and Systematics Laboratory
https://www.discoverlife.org/mp/20l?id=BBSL802531
iNaturalist.org: iNaturalist research-grade observations
https://www.discoverlife.org/mp/20l?id=INAT3967713
iNaturalist.org: iNaturalist research-grade observations
https://www.discoverlife.org/mp/20l?id=INAT4245327
iNaturalist.org: iNaturalist research-grade observations
https://www.discoverlife.org/mp/20l?id=INAT552487
